# SSN2V: unsupervised OCT denoising using speckle split

**DOI:** 10.1038/s41598-023-37324-5

**Published:** 2023-06-27

**Authors:** Julia Schottenhamml, Tobias Würfl, Stefan B. Ploner, Lennart Husvogt, Bettina Hohberger, James G. Fujimoto, Andreas Maier

**Affiliations:** 1 Pattern Recognition Lab, Friedrich-Alexander-Universität Erlangen-Nürnberg, Erlangen, Germany; 2 Research Laboratory of Electronics, Department of Electrical Engineering and Computer Science, Massachusetts Institute of Technology, Cambridge, MA USA; 3 Department of Ophthalmology, Universitätsklinikum Erlangen, Friedrich-Alexander-Universität Erlangen-Nürnberg, Erlangen, Germany

**Keywords:** Computer science, Software

## Abstract

Denoising in optical coherence tomography (OCT) is important to compensate the low signal-to-noise ratio originating from laser speckle. In recent years learning algorithms have been established as the most powerful denoising approach. Especially unsupervised denoising is an interesting topic since it is not possible to acquire noise free scans with OCT. However, speckle in in-vivo OCT images contains not only noise but also information about blood flow. Existing OCT denoising algorithms treat all speckle equally and do not distinguish between the noise component and the flow information component of speckle. Consequently they either tend to either remove all speckle or denoise insufficiently. Unsupervised denoising methods tend to remove all speckle but create results that have a blurry impression which is not desired in a clinical application. To this end we propose the concept, that an OCT denoising method should, besides reducing uninformative noise, additionally preserve the flow-related speckle information. In this work, we present a fully unsupervised algorithm for single-frame OCT denoising (SSN2V) that fulfills these goals by incorporating known operators into our network. This additional constraint greatly improves the denoising capability compared to a network without. Quantitative and qualitative results show that the proposed method can effectively reduce the speckle noise in OCT B-scans of the human retina while maintaining a sharp impression outperforming the compared methods.

## Introduction

Optical coherence tomography (OCT) is an imaging modality^1^ for non-invasive, in-vivo, three-dimensional imaging of the retina in micrometer scale. However, OCT images are degraded by a high amount of speckle noise which renders the interpretation of fine details in these images a challenging task. The underlying problem is that photons are not perfectly reflected from the tissue but may also be scattered and detected at different positions or be scattered and not detected at all^2^. But not all speckle in an OCT scan can be regarded as uninformative noise. In in-vivo imaging, moving particles in blood vessels cause signal variation over time, depending on the position, shape and orientation of the particles and therefore lead to speckle that contains information about this motion. There are different approaches for the denoising of OCT B-scans which can be roughly separated into two groups: single-frame methods and multi-frame methods. In multi-frame algorithms, multiple scans are acquired at the same physical position and then registered and averaged^3–5^. The drawback of this approach is that several scans are needed, which increases the scan time. This is especially problematic when scanning elderly patients or patients with low visual acuity as they tend to have worse fixation introducing motion artifacts. Moreover the scans have to be registered introducing registration errors. The single-frame methods can be further divided into unsupervised and supervised methods. The later nowadays are mostly based on deep learning approaches using CNNs^6–10^ and loss functions based on GANs^11–13^ that require a ground truth for training. A problem with this approach is that it is not possible to acquire noise-free OCT scans on a physical measurement system. Therefore multiple scans are taken at the same physical position and are, as in the multi-frame case, averaged to create a ground truth. Consequently the same shortcomings as in the multi-frame case apply. For unsupervised single-frame methods standard approaches, like median or bilateral filtering and more sophisticated ones based on wavelets^14–16^ or variations of non-local means^17,18^ exist. These methods usually still preserve a large amount of the speckle or are computationally expensive. One property that all of the denoising methods have in common is that they do not differentiate between speckle that contains information and uninformative speckle typically referred to as noise. Consequently if one of these algorithms would be able to perfectly remove all speckle it would also remove the informative speckle as well. This is also accompanied by the impression of blurry images which is not desired in clinical applications. We therefore propose a two-step unsupervised approach for OCT denoising called speckle split Noise2Void (SSN2V), which, in contrast to other denoising methods, is able to preserve the speckle that contains flow information. This is achieved by incorporating known operators^19^ into the network. This additional constraint greatly improves the denoising capability in contrast to a N2V network without the constraint. SSN2V is able to denoise the OCT B-scans and retain a sharper and less blurry impression of the results than the compared methods.

## Methods

**Figure 1:**
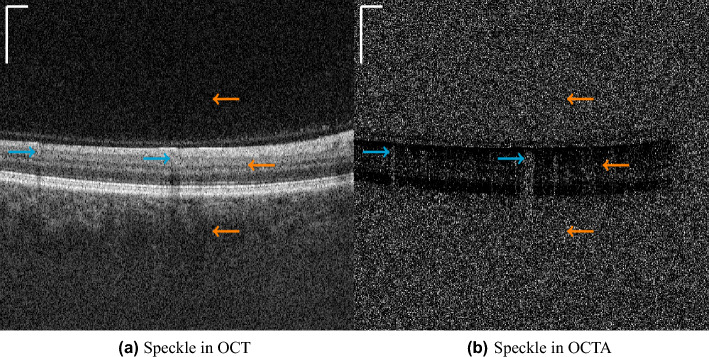
Examples for speckle that contains flow information (blue arrows) originating from scattering of photons at moving blood cells and speckle noise (orange arrows) originating from scattering of photons in the vitreous humor (upper arrow), the static tissue (middle arrow) or the choroid (lower arrow). The scale bars in the top left corners indicate $360~\mu m$.

As introduced earlier, the speckle in OCT scans is not only noise but also originates from moving particles in the scanned specimen. Thus, this speckle provides information about the blood flow, which is visualized in OCT angiography (OCTA). For the computation of an OCTA B-scan, multiple OCT B-scans are acquired at the same physical position within a short time interval and a variation metric is computed among them. In the ideal case, where there is no speckle noise, all photons are perfectly reflected and detected, causing the intensities in OCT B-scans to be the same as in static tissue, which in turn leads to no signal in these locations in the OCTA B-scans. But since in reality there is also some scattering and minimal motion in static tissue, there is also a variation in intensities leading to uninformative speckle noise. Consequently OCT and OCTA images contain speckle that is uninformative, which we also will refer to as noise in the paper, and speckle that contains information on the blood flow, as depicted in Fig. 1.

**Figure 2:**
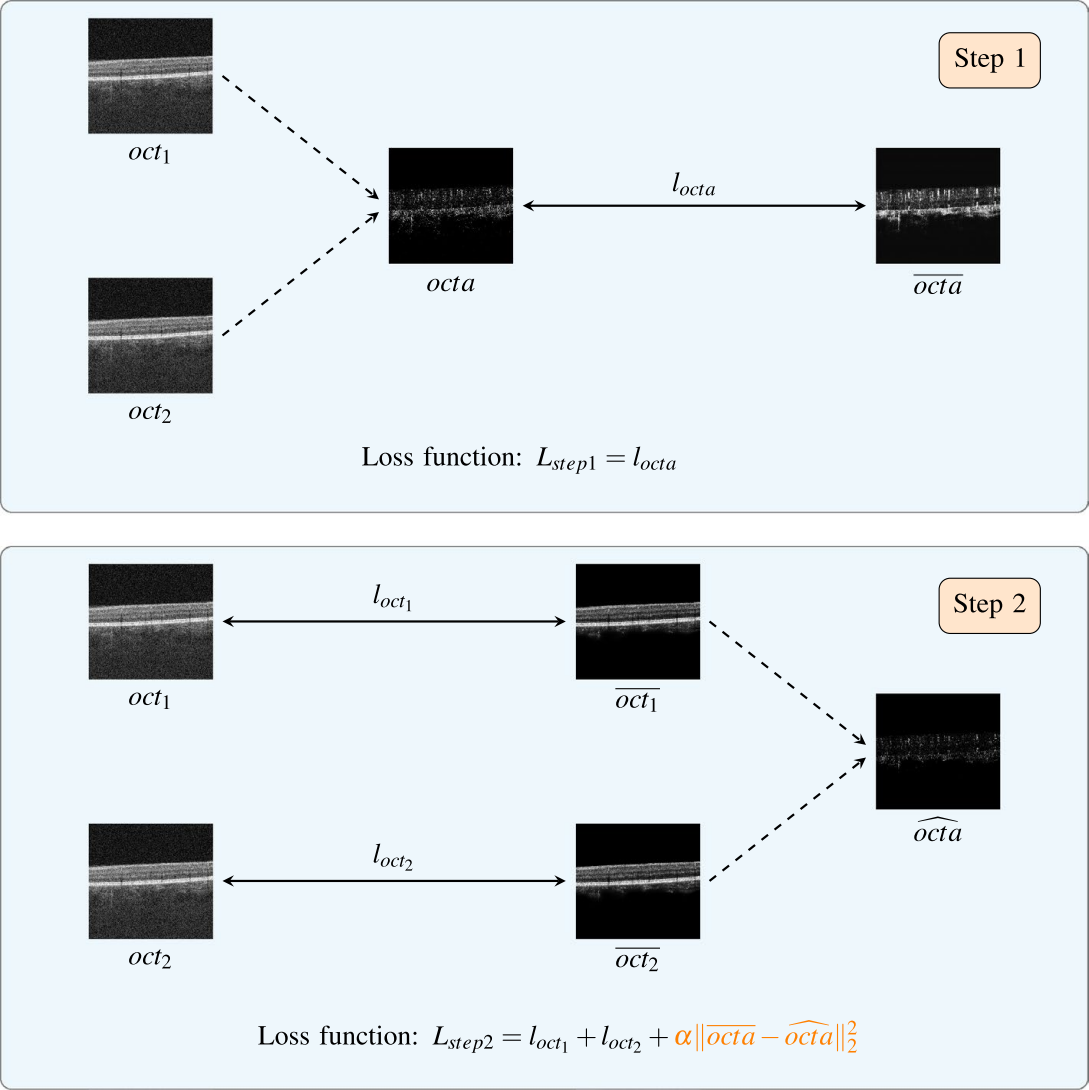
Depiction of the construction of the loss functions in our proposed 2-step procedure. In the first step an OCTA B-scan (*octa*) is computed from two OCT B-scans ($oct_1$, $oct_2$) and is denoised ($\overline{octa}$) using a U-net (U-net$_{OCTA}$). The loss function for this first step ($L_{step1}$) is the loss function from the N2V scheme ($l_{octa}$). In the second step, a new U-net is introduced for denoising the OCT B-scans (U-net$_{OCT}$). Two OCT B-scans ($oct_1$, $oct_2$) are denoised ($\overline{oct_1}$, $\overline{oct_2}$) separately using the same U-net (U-net$_{OCT}$). This leads to two losses from the N2V scheme, one for each denoised OCT B-scan ($l_{oct_{1}}$ and $l_{oct_{2}}$). From these denoised OCT B-scans, an OCTA B-scan ($\widehat{octa}$) can then be computed. This OCTA B-scan can then be used as a constraint that it should remain as close as possible to the denoised OCTA B-scan from the first step ($\overline{octa}$). This ensures that the speckle information is kept. The total loss for the second step ($L_{step2}$) that is used to train the U-net of this step (U-net$_{OCT}$) is then a combination of the two losses from the N2V scheme ($l_{oct_{1}}$ and $l_{oct_{2}}$) and the OCTA constraint ($\alpha \Vert \overline{octa} - \widehat{octa} \Vert _2^2$). Since the constraint contains a fully differentiable OCTA computation block it can be incorporated into an end-to-end training.

In this paper, we introduce the novel concept that our proposed denoiser should, in addition to reducing uninformative noise, also preserve OCTA flow information. Consequently the algorithm needs to be able to separate uninformative speckle noise from the informative speckle signal and only remove the former. With OCTA, the speckle information and the speckle noise can be better separated, because the vessel information is spatially clustered in those images. Therefore, we propose a two-step algorithm: In the first step, OCTA images are denoised in an unsupervised manner. In a second step, these denoised OCTA images are then incorporated into a constraint for the OCT denoising. Because of that constraint and since the OCTA images are denoised, the OCT denoising network in the second step learns to keep the speckle information that is necessary to still be able to compute the OCTA signal while denoising the OCT B-scans. A deptiction of the principles of our 2-step procedure is outlined in Fig. 2.

### Step 1: unsupervised OCTA denoising

In the first step we take the OCTA B-scan (*octa*) computed from two noisy OCT B-scans ($oct_1$, $oct_2$) and denoise it unsupervised ($\overline{octa}$). To calculate the OCTA B-scans in the first step (*octa*), full-spectrum decorrelation^20^ is used, according to:

1 $$\begin{aligned} octa(x,z) = \frac{(oct_1(x,z) - oct_2(x,z))^2}{oct_1(x,z)^2 + oct_2(x,z)^2} \end{aligned}$$

**Figure 3:**
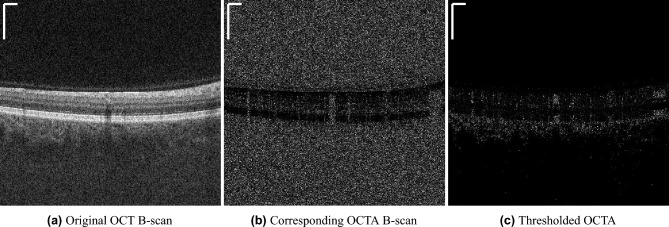
The intensity values from the OCT B-scan (**a**) can be used to threshold the corresponding OCTA B-scan (**b**) in regions with low OCT-signal. The result is a thresholded OCTA B-scan (**c**) where the speckle noise is removed in these areas. The scale bars in the top left corners indicate $360~\mu m$.

As can be seen in Fig. 3b there is a lot of speckle noise in the non-retinal regions. These regions concur with regions in the OCT B-scan that show low signal (Fig. 3a).

Jia et al.^20^ assume that speckle caused by blood cell movement cannot be assessed accurately in these low signal regions, because the speckle noise is dominating the flow information. Consequently, the OCT B-scans can be used to threshold the original OCTA B-scans and thus remove the high signal in regions with low reflection which are known to be avascular, like the vitreous, or uncertain regions, like the deeper choroid. This thresholding operation is described in more detail in section ’4.1 Removal of high decorrelation generated by background noise’ in the aforementioned work by Jia et al.^20^. The results are thresholded OCTA B-scans as depicted in Fig. 3c where only speckle in regions with higher OCT signal is left. For the unsupervised denoising, we use a Noise2Void (N2V)^21^ training scheme with a U-Net of depth three as backbone. Using N2V, a single noisy input frame is used as input for the network and as target at the same time. The training is done using the blind-spot network, as described by the authors of the N2V paper^21^, where the center pixel in the receptive field is masked in order to prevent the network from learning the identity. The training was done on a B-scan basis and from each B-scan a patch of size $100 \times 100$ pixels was randomly cropped out of the image. The learning rate was set to 0.0001 and was reduced on plateau, with a patience of 10 and a reduction factor of 0.5, with Adam as optimizer. As loss function the mean squared error (${{\,\mathrm{MSE_{N2V}}\,}}$), as introduced in^21^, between the prediction ($\overline{octa}$) and the target (*octa*) for the masked pixels was used leading to the following loss for the first step:

2 $$\begin{aligned} L_{step 1} = {{\,\mathrm{MSE_{N2V}}\,}}(\overline{octa}, octa) \end{aligned}$$

### Step 2: unsupervised OCT denoising using an OCTA constraint

In the second step, we train a second U-Net with the N2V training scheme to denoise OCT B-scans. The U-Net network architecture and training parameters are the same as in the first step. Firstly, two corresponding OCT B-scans ($oct_1$ and $oct_2$) are denoised separately by the same U-Net. From these two denoised OCT scans ($\overline{oct_1}$, $\overline{oct_2}$) the OCTA image ($\widehat{octa}$) is computed. This OCTA B-scan can then be used as a constraint that it should remain as close as possible to the denoised OCTA B-scan from the first step ($\overline{octa}$). This ensures that the speckle information is kept. In this work we chose the constraint to be the $L^2$-norm of the difference between $\widehat{octa}$ and $\overline{octa}$. The computation of the OCTA B-scans is, as in the first step, done using full-spectrum decorrelation:

3 $$\begin{aligned} \widehat{octa}(x,z) = \frac{(\overline{oct_1}(x,z) - \overline{oct_2}(x,z))^2}{\overline{oct_1}(x,z)^2 + \overline{oct_2}(x,z)^2} \end{aligned}$$

This time the retina thresholding is not applied. This is because if the thresholding would be applied to the reconstructed OCTA B-scans, the pixel values in low OCT-signal regions would be set to zero and therefore not contribute to the constraint in the loss. As a consequence all denoising performed in those low OCT-signal regions would not influence the loss and therefore the network would not learn to denoise in the background. This constraint is added to the two losses obtained from the N2V scheme from densoising the two corresponding OCT B-scans, which leads to the following loss for the second step:

4 $$\begin{aligned} L_{step 2} = {{\,\mathrm{MSE_{N2V}}\,}}(\overline{oct_1}, oct_1) + {{\,\mathrm{MSE_{N2V}}\,}}(\overline{oct_2}, oct_2) + \alpha \cdot \Vert \overline{octa} - \widehat{octa} \Vert _2^2 \end{aligned}$$

**Figure 4:**
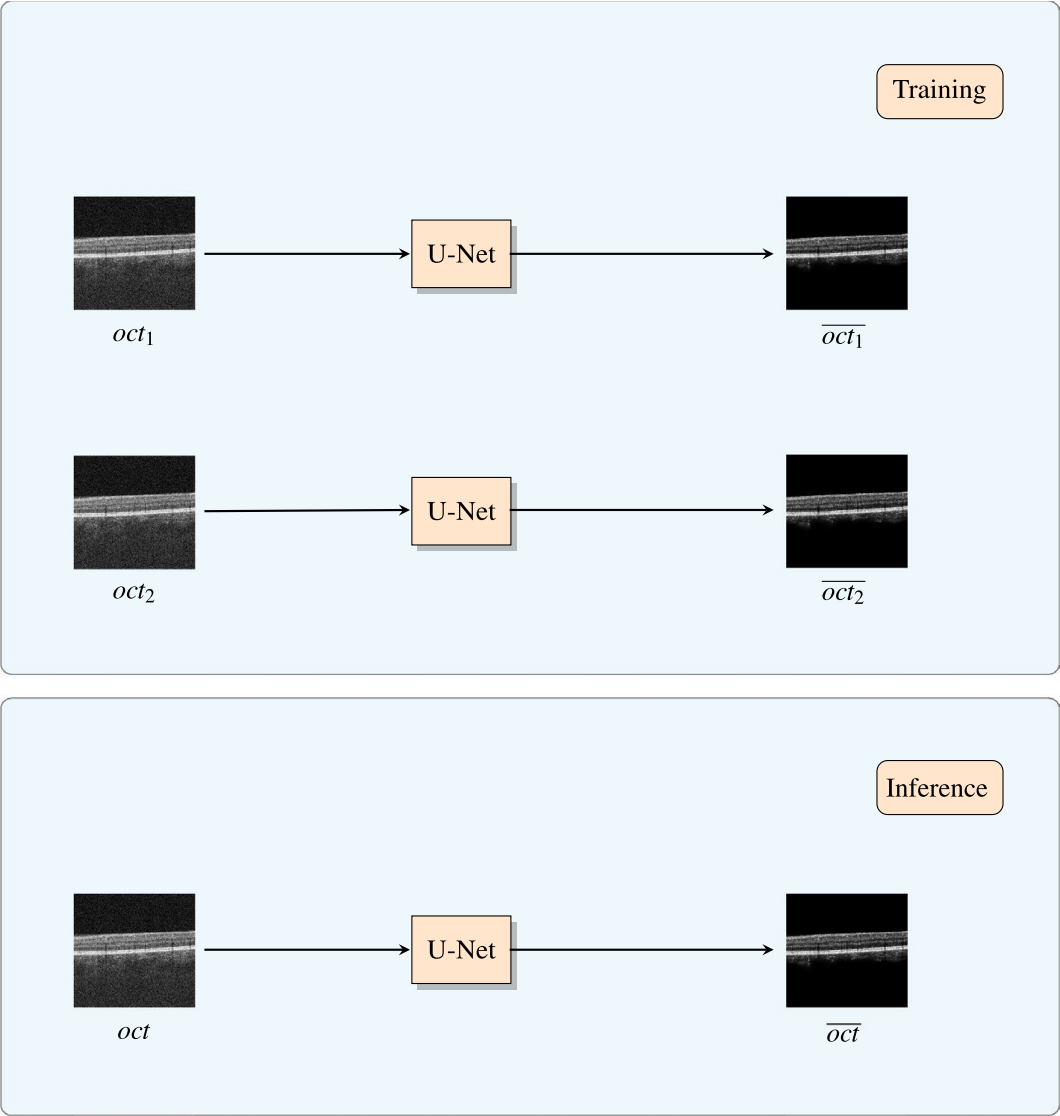
At training time the loss function depends on denoising two OCT B-scans. So the same U-Net is used to separately denoise each of the two images. In contrast to that, at inference time, the loss function does not need to be computed anymore. Consequently, since the network in the training phase is used separately on a single OCT B-scan, a single OCT B-scan can be denoised on its own in this phase.

Since $\overline{octa}$ is constant in this second step, the constraint only depends on the OCTA B-scan computed from the denoised OCT B-scans $\overline{oct_1}$ and $\overline{oct_2}$. This OCTA computation can be implemented fully differentiable. Therefore, it can be integrated into the constraint of the loss function and into the network as a known operator^19^. This enables an end-to-end training of the U-Net in this second step. The parameter $\alpha$ is for the regularization of the constraint term. The smaller it is, the smaller the effect of the constraint and the closer the denoised results are to denoised images produced with N2V. The higher it is, the more focus will be set on reconstructing the OCTA signal and less on the denoising. In this work $\alpha$ was set to 1. This value was chosen because then the denoising term and the OCTA reconstruction term have equal contribution in the resulting loss. Another subtlety is that the denoising is performed on the linear-scale OCT B-scans and the logarithmic transformation commonly used for display is calculated afterwards. This is necessary because OCTA B-scans are computed from non-logarithmic images. So the denoised OCT B-scans ($\overline{oct_1}$, $\overline{oct_2}$) need still to be non-logarithmic, because otherwise the reconstructed OCTA B-scans ($\widehat{octa}$) for the constraint could not be computed. Finally note that, since the OCT B-scan denoising U-Net was trained with single OCT B-scans as input and the loss function which depends on two OCT B-scans does not need to be computed anymore during inference, the denoising can be performed on a single OCT image without the need to acquire multiple repeated scans. This concept is displayed in Fig. 4.

## Evaluation

### Data

In total 108 scans from 39 patients were identified retrospectively. A variety of pathologies were included to be able to evaluate the performance of the proposed method in regions with non-healthy retinal structure, like drusen, hemorrhage, area of geographic atrophy, etc. The scans were divided into a training and a test set whereupon scans from the same patient were assigned exclusively to only one of them. For validation purposes and algorithm parameter selection, B-scans were randomly sampled from the training set. The exact partitioning can be found in Table 1.

**Table 1:** Distribution of volumes per pathology (number of patients) for the training and test set.

Set	Field sizes	Pathology								Total
		CNV	DM no DR	early AMD	GA	NAION	Normal	NPDR	PDR	
Training set	3 $\textrm{mm}$	2 (1)	4 (2)	2 (1)	4 (1)	4 (1)	32 (6)	8 (4)	14 (5)	70 (21)
	6 $\textrm{mm}$	2 (1)	2 (1)	0 (0)	10 (4)	0 (0)	4 (2)	2 (1)	0 (0)	20 (9)
Test set	3 $\textrm{mm}$	0 (0)	2 (1)	0 (0)	0 (0)	0 (0)	2 (1)	2 (1)	2 (1)	8 (4)
	6 $\textrm{mm}$	2 (1)	2 (1)	0 (0)	2 (1)	0 (0)	2 (1)	2 (1)	0 (0)	10 (5)
Total	3 $\textrm{mm}$	2 (1)	6 (3)	2 (1)	4 (1)	4 (1)	34 (7)	10 (5)	16 (6)	78 (25)
	6 $\textrm{mm}$	4 (2)	4 (2)	0 (0)	12 (5)	2 (1)	6 (3)	4 (2)	0 (0)	30 (14)

The test set contains 18 volumes from nine patients. Since each volume consists of 500 B-scans, these are 9000 OCTA B-scans and 18000 OCT B-scans in total. OCT scans were acquired with a 400 kHz SS-OCT system with 500 A-scans at 500 distinct retinal locations. The A-scans consisted of 480, 465 or 433 pixel. Each B-scan was repeated two times in order to be able to compute an OCTA B-scan. The scans were centered on the fovea and $3\times 3$ mm or $6\times 6$ mm areas were imaged using a raster scan protocol. The SS-OCT system has a pixel spacing of $\sim$4.5 $\mu m$ in the axial (*z*) coordinate, and $\sim$6 $\mu m$ or $\sim$12 $\mu m$ in the transverse (*x* and *y*) coordinates. Per patient at least one orthogonal x-fast and y-fast OCT dataset was acquired. Accounting for the galvanometer duty cycle, the interscan time between repeated B-scans was $\sim$1.5 ms. OCTA images were computed using amplitude decorrelation according to Jia et al.^20^.

### Experiments

In this study, we investigated the denoising performance of different algorithms for the OCT B-scans in the second step of our algorithm. Here, our proposed SSN2V method was compared to four unsupervised methods, namely BM3D^22^, TV^23^, WNNM^24^ and N2V^21^. The U-Net trained with the plain N2V algorithm without the OCTA reconstruction constraint was added for comparison in order to investigate the influence of the constraint. The hyperparameters of each of the algorithms were tuned on the validation set (BM3D: sigma 0.05, TV: weight 0.75, WNNM: variance 20, N2V: see subsection “Step 1: Unsupervised OCTA denoising”, SSN2V: see subsection “Step 2: Unsupervised OCT denoising using an OCTA constraint”). Since no ground truth and no noise model is available for this task we conducted a user study where a senior ophthalmologist with more than 10 years of experience in ophthalmology was shown an OCT B-scan and the anonymized results of the five denoising algorithms in order to evaluate our approach. The expert had to rate every image with a number between 0 (does not approve of the image quality) to 5 (approves the image quality). This process was done for 35 randomly chosen B-scans and reflects the opinion of clinical experts, which in this case is the most significant marker. To further analyse these scores, analysis of variance (ANOVA) was used to see if there are statistically significant differences between the methods. The statistical analysis was performed using SPSS version 28 (IBM Corp. Released 2021. IBM SPSS Statistics for Windows, Version 28.0. Armonk, NY: IBM Corp.). *P* values less than 0.05 were considered to be statistically significant and Bonferroni adjustment was used to account for multiple comparisons.

## Results and discussion

**Figure 5:**
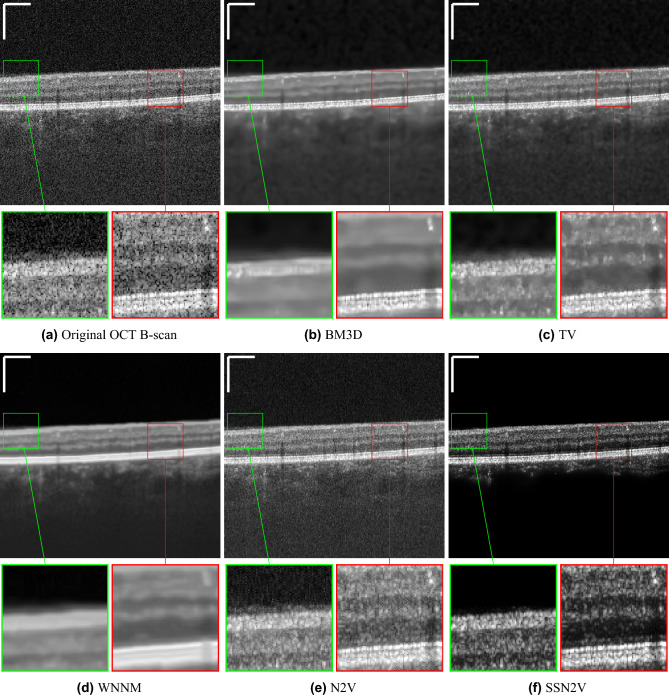
Qualitative examples for the denoising of OCT B-scans ($\overline{oct_x}$). (**a**) Shows the original OCT B-scan while the following subfigures show the results for the different denoising algorithms. Images are each displayed in the range of from their minimal to their maximal intensity. The scale bars in the top left corners indicate $360~\mu m$.

Visual impressions for the denoising performance of the different algorithms can be seen in Fig. 5. The methods show different characteristics: First of all, the N2V approach without constraint only has a very weak denoising performance and lots of background noise remains. The BM3D method creates a more blurry image where the noise structure in the background can still be seen. The WNNM algorithm creates an even blurrier image. While in the other algorithms, multiple bright bands can be distinguished in the photoreceptor layers and the retinal pigment epithelium (RPE), only one single wide band appears in the WNNM result. Moreover, the contrast between the retinal tissue and the vessel locations is diminished, making it hard to locate them in the scan. The TV method is a compromise between BM3D/WNNM and N2V. It keeps more background noise than BM3D and WNNM but is also less blurry. In contrast to the other four algorithms, our proposed SSN2V algorithm is able to distinguish the background from the retinal tissue and reduces the amount of speckle noise. Moreover the area covered by the retina is denoised, the retinal layers can be clearly distinguished and the vessel locations are well visible.

**Figure 6:**
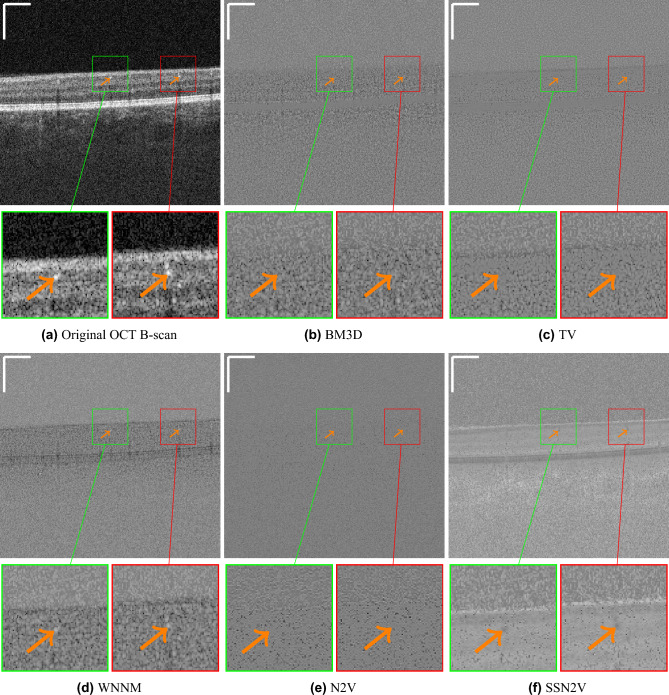
Qualitative examples for residual images from the same B-scan as in  Fig. 5.  Fig. 6a shows the original OCT B-scan while the following subfigures show the residual images for the different denoising algorithms from the original OCT B-scan. The green and red boxes highlight regions with vessels that are highlighted by the orange arrows. They show that the SSN2V algorithm is the only one removing less speckle at the vessel locations compared to the surrounding tissue, while BM3D, TV and N2V make no distinction and WNNM even removes more speckle at vessel locations. This indicates that it is capable of distinguishing the informative from the uninformative speckle and only removes the latter. The scale bars in the top left corners indicate $360~\mu m$.

Figure 6 shows the residual images from an original OCT B-scan and the denoised versions of the different algorithms. These images reveal that the proposed SSN2V algorithm is most effective in removing speckle noise. Moreover, within blood vessels, less speckle is removed than in the surrounding tissue. This behaviour is not shown by all other algorithms. This demonstrates that the algorithm is able to differentiate between informative and uninformative speckle and only removes the latter.

**Figure 7:**
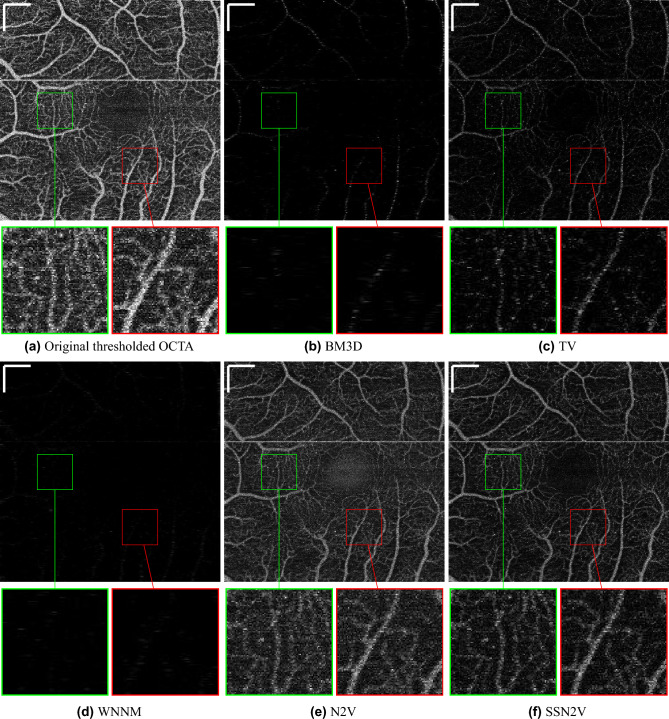
Qualitative examples for the enface projection of OCTA B-scans computed from denoised OCT B-scans (enface $\widehat{octa}$). (**a**) Shows the original projection of OCTA B-scans while the following subfigures show the results for the different denoising algorithms. In order to be able to qualitatively judge the similarity of these images, they are displayed using the same intensity windowing. The green and red boxes show magnified views from the same locations indicated in the images. The horizontal white line is a motion artifact. The scale bars in the top left corners indicate $360~\mu m$.

 To validate the preservation of flow-related components qualitatively, Fig. 7 shows en face OCTA images from the original and the denoised OCT scans (en face $\widehat{octa}$). They are in line with the observations made from the denoised OCT B-scans in Fig. 5. In the case of BM3D and WNNM nearly no or only a relatively weak OCTA signal can be recovered. Since these two methods favour smoother results, they removed all speckle and, in removing the speckle noise, also removed nearly all the speckle information. Moreover it can be seen that the OCTA image computed from the N2V-denoised OCT B-scans is very close to the original OCTA B-scan. This can also be explained by the fact that the N2V algorithm has a very limited denoising capability on OCT and therefore little speckle (noise and information) is erased. Again, in these images, the TV method is a compromise because it reduces most speckle noise but also keeps only little speckle information of the retinal vasculature leading to only weak signal in areas of large vessels. For the proposed SSN2V method we can see that the speckle noise in the intercapillary areas is reduced while all the vessels are still clearly visible. This again also demonstrates the ability to discriminate between informative and uninformative speckle.

**Figure 8:**
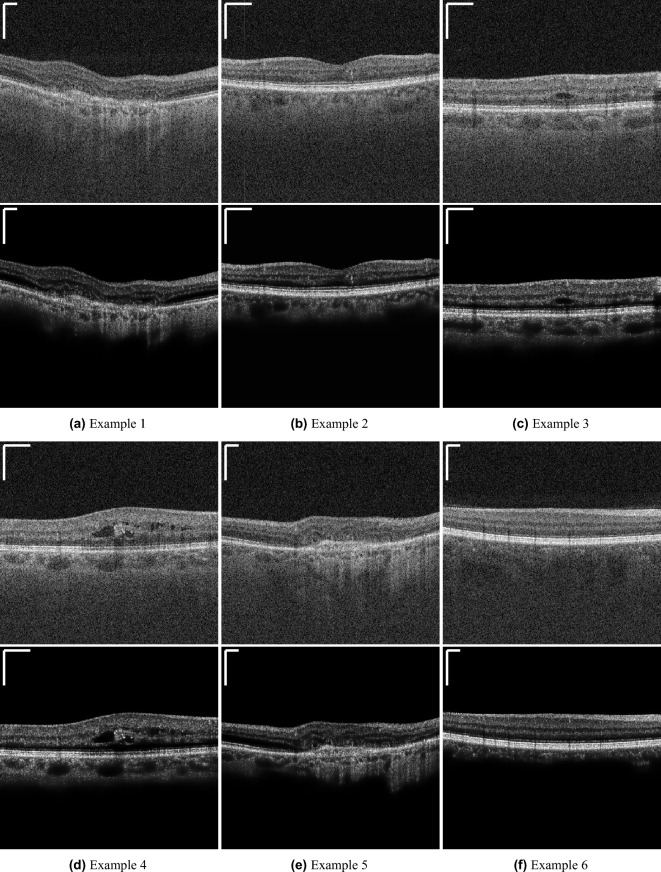
Qualitative examples for the denoising result of the SSN2V algorithm. The subfigures show the original input OCT images (top) and the denoised result from the SSN2V algorithm (bottom). The scale bars in the top left corners indicate $360~\mu m$.

More qualitative denoising results of the SSN2V algorithm are shown in Fig. 8. These results show, that the proposed SSN2V algorithm also works on different pathological cases and features.

**Figure 9:**
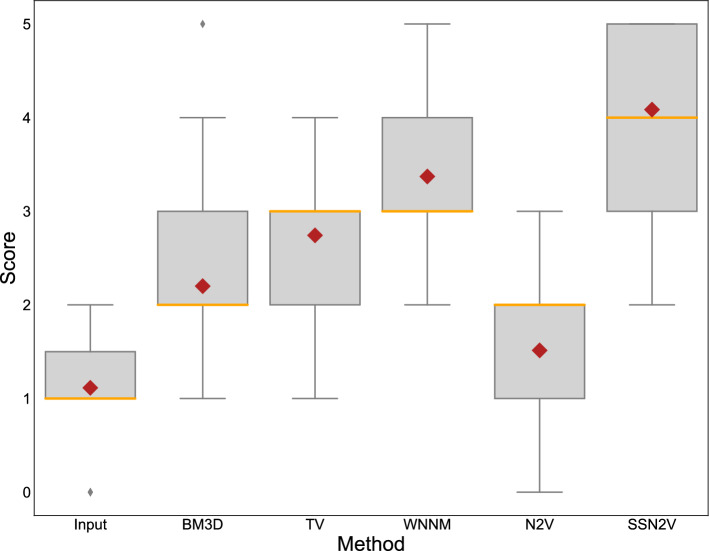
Boxplots for the scores from the ophthalmologist for the different methods. The orange line indicates the median values while the red diamond the mean value.

The mean and standard deviation of the scores distributed by the ophthalmologist are given in Table 2. The boxplots for these values can be found in Fig. 9. The input image achieves the lowest scores, as expected. Only slightly higher scores were given the N2V approach without using the constraint. This is in accordance with the qualitative examples where this method did not seem to remove a lot of speckle noise. Then the BM3D, TV and WNNM methods perform in ascending order. This is also in accordance with the visual impression shown earlier, where these methods have a varying degree of trade-off between not denoising a lot and creating blurry results. Our proposed SSN2V algorithm has the unique combination of denoising the images and preserving the flow-related speckle and is preferred by expert ophthalmologists in a clinical setting. The statistical analysis revealed that all differences are statistically significant except between the input images and N2V and between BM3D and TV.

One disadvantage of the SSN2V algorithm is that at training time it needs two OCT B-scans while all other unsupervised algorithms only need a single OCT B-scan. This is owed to the circumstance that during training two OCT B-scans are needed to calculate the loss function. However, the same U-net is applied to each of the two OCT B-scans separately and therefore the network itself only depends on one OCT B-scan as input. Consequently this drawback is only present at training time when the loss function is needed. For inference, when the network has been trained and the loss function does not need to be computed anymore, a single OCT B-scan can be denoised using the trained SSN2V network without the need for a second one. In contrast to supervised denoising methods, that generate their ground truth through averaging, only two consecutive OCT B-scans are required for the proposed SSN2V approach for the training which would not be enough for ground truth generation in the supervised case. This has the advantage that no repeated acquisitions and motion correction is needed, such that data that was acquired for OCTA computation can be used retrospectively as training data. Moreover, the concept of incorporating OCTA infomration into the loss function is a general concept and could also be implemented and used for supervised methods if ground truth data is available. Overall, the results show that while other algorithms blur the OCT B-scan to a varying but uniform degree, the SSN2V approach denoises the image while keeping the sharp borders between the different retinal layers. Moreover, when inspecting the reconstructed OCTA images, the results are close to the original input while even appearing denoised. All compared algorithms removed the noise and information components of speckle similarly. Despite only a limited number of other denoising methods were used for comparison, they are suitable and sufficient representatives to justify the claim: since no other denoising method was designed to differentiate between speckle noise and speckle information we assume that they all will behave in the same manner and show a varying OCT denoising capability but will remove all speckle equally and thus will fail to achieve both goals at the same time. This combination of denoising while preserving the speckle information is a unique property of our method and will most likely not be achieved with any other denoiser at the moment.

**Table 2:** Mean ± the standard deviation of the scores given by the clinical expert in the user study. The best score is indicated in bold.

	Input	BM3D	TV	WNNM	N2V	SSN2V
Score	1.11 ± 0.622	2.2 ± 0.92	2.74 ± 0.73	3.37 ± 0.93	1.51 ± 0.77	**4.09 ± 0.87**

## Conclusion

In this paper we proposed a novel denoising concept for OCT B-scans which consists of separating informative speckle from uninformative noise and remove only the latter. Based on this paradigm we introduced a novel, differentiable OCTA computation block as a known operator into our network which made it possible for the network to distinguish between speckle noise and speckle information. We presented a fully unsupervised denoising method for OCT B-scans (SSN2V) which performs similar or better to existing denoising methods but outperforms any classical method in the sense that it preserves OCTA information. Moreover it is able to generate sharper-looking results. This was demonstrated by a user study, where the expert ophthalmologist gave the highest scores to the proposed method in a clinical setting. This separation of speckle noise and speckle information is not possible with any other denoising algorithm at the moment.

## Data Availability

The dataset analysed during the current study is not publicly available due to general data privacy regulations but is available from the corresponding author on reasonable request.
